# Exploring Novel Therapeutic Approaches for Depressive Disorders: The Role of Allopregnanolone Agonists

**DOI:** 10.7759/cureus.44038

**Published:** 2023-08-24

**Authors:** Najeeha Ahmad Bhatti, Anna Jobilal, Kainat Asif, Mateo Jaramillo Villegas, Priyanka Pandey, Ayzal Noor Tahir, Neeharika Balla, Maryoreht Paola Arellano Camargo, Sana Ahmad, Janvi Kataria, Zain U Abdin, Muhammad Ayyan

**Affiliations:** 1 Internal Medicine, Noonu Atoll Hospital, Manadhoo, MDV; 2 Internal Medicine, Sri Ramaswamy Memorial Medical College Hospital and Research Centre, Kattankulathur, IND; 3 Internal Medicine, Dr. Ruth K. M. Pfau Civil Hospital, Karachi, PAK; 4 Internal Medicine, Universidad CES Escuela de Medicina, Medellín, COL; 5 Anatomical Sciences, Hind Institute of Medical Sciences, Sitapur, IND; 6 Psychiatry, Akbar Niazi Teaching Hospital, Islamabad, PAK; 7 Internal Medicine, Maharajah’s Institute of Medical Sciences, Vizianagaram, IND; 8 Internal Medicine, University Hospital Dr. Alfredo Van Grieken, Coro, VEN; 9 Psychiatry, TIME Organization Inc, Baltimore, USA; 10 Internal Medicine, DY Patil University, Mumbai, IND; 11 Family Medicine, IMG Helping Hands, Chicago, USA; 12 Internal Medicine, Mayo Hospital, Lahore, PAK

**Keywords:** major depressive disorder, zuranolone, brexanolone, postpartum depression, major depressive disorder (mdd), narrative review

## Abstract

Depressive disorders are caused due to the impaired functioning of important brain networks. Recent studies have also shown that it is caused by a significant reduction in the levels of allopregnanolone, which is a progesterone metabolite. Newer treatment modalities are now focusing on the usage of neuroactive steroids, such as allopregnanolone, in various depressive disorders. Our aim was to provide a comprehensive literature review on the clinical aspects of the allopregnanolone agonists brexanolone and zuranolone with reference to the physiological role of allopregnanolone. Brexanolone was approved by the FDA in 2019 for the treatment of postpartum depression and has greatly influenced further research into potential drugs such as zuranolone, which is currently undergoing phase 3 of clinical trials. Although these drugs exhibit improvement in symptoms of depressive disorders along with notable side effects, further research is required for their future clinical use.

## Introduction and background

Depressive disorders are widespread and incapacitating conditions associated with significantly reduced performance in daily life and overall well-being. They also lead to an increased rate of occurrence of other medical conditions and a higher mortality rate [1]. Over the course of recent decades, substantial strides have been made in the scientific exploration and advancement of treatment modalities for depressive disorders across various healthcare contexts encompassing primary care. This progress has paved the way for a diverse array of antidepressant medications and psychotherapeutic modalities to become widely accessible [2].

Our current understanding of the etiology and therapeutic interventions for depression and stress-related disorders remains incomplete, as there are still many aspects that have not been fully elucidated. However, recent investigations conducted on patients with depression and animal models have started to provide encouraging results. These studies indicate that depression and chronic exposure to stress lead to the deterioration of neurons in specific brain regions associated with depression, both in the limbic system and the cortex [3,4]. Additionally, brain imaging studies reveal notable changes in connectivity and network functioning within the brains of individuals with depression [3].

Depression should be viewed as a spectrum comprising various interconnected conditions, ranging from postpartum depression (PPD) to major depressive disorder (MDD). Many forms of depression have been described in the literature, including minor depressive disorder, MDD, mixed depression, brief depressive disorder, and dysthymic disorder [5]. MDD is distinguished by the presence of prevalent feelings of sorrow and despair, decreased ability to derive pleasure (anhedonia), lowered motivation, cognitive challenges, and physiological manifestations affecting essential bodily functions [6-8].

Various treatment methods are available for addressing depression, encompassing both pharmacological and non-pharmacological options. Non-pharmacological approaches include electroconvulsive therapy, transcranial magnetic stimulation, and psychotherapy. Psychotherapy has demonstrated efficacy in mitigating depressive symptoms and improving the overall well-being of individuals afflicted with depression [9,10].

Pharmacological treatment is mainly based on the neurotransmitter theory of depression and includes drug classes like selective serotonin reuptake inhibitors (SSRIs), serotonin and norepinephrine reuptake inhibitors (SNRIs), tricyclic antidepressants, monoamine oxidase inhibitors (MAOIs), and some atypical antidepressants [11].

Most antidepressants were serendipitously discovered many years ago. While the majority of antidepressants are widely acknowledged as being safe and effective, there are certain limitations associated with their use. These include a delay in their effectiveness, typically around two weeks, as well as side effects that can impact adherence to treatment [12]. Furthermore, less than half of all individuals with depression achieve full recovery despite receiving optimized treatment, which involves experimenting with various medications both with and without concurrent psychotherapy. Another limitation of current treatment is that around 30% of individuals experiencing depression are labeled as treatment-resistant due to their inability to derive any positive effects from two or more medications targeting antidepressant efficacy [13,14]. Only a small percentage of individuals who benefit from these treatments experience remission, rendering the monoamine hypothesis for MDD inadequate and highlighting the unmet need for more effective antidepressant treatments [15]. Hence, it is widely accepted that the antidepressant drugs prescribed at present do not directly target the specific neurochemical systems implicated in MDD. Rather, these medications act on alternative neuronal pathways that hold a more central position in MDD's underlying mechanisms [15].

In the year 2019, the Food and Drug Administration (FDA) granted approval for two newly developed antidepressant medications. These include esketamine, which is specifically intended to address treatment-resistant depression, and brexanolone, designed to combat PPD. Furthermore, esmolamine, a derivative of ketamine (an anesthetic substance), has also obtained FDA approval as a therapeutic option for patients suffering from refractory depression [16]. Thus far, although several potential medications have yet to receive FDA approval, they represent significant advancements in the field of antidepressant development and hold promise for future clinical use. Potential future uses of clinical compounds may involve the modification and utilization of various substances. Examples include compounds that consist of dextromethorphan, which acts as a non-selective antagonist for N-methyl-D-aspartate (NMDA) receptors. Additionally, sarcosine, known as N-methylglycine and functioning as an inhibitor for glycine reuptake, is another compound with therapeutic potential. Modulating AMPA (α-amino-3-hydroxy-5-methyl-4-isoxazole propionate) receptors or metabotropic glutamate receptors (mGluRs) is another avenue worth exploring for modifying these substances to enhance their efficacy in future clinical settings [17]. Evidence of the therapeutic effectiveness of allopregnanolone in PPD provides additional support to the GABAergic deficit hypothesis in MDD. These data suggest that ligands for neuroactive steroid sites, such as allopregnanolone, could represent a novel class of medications for affective disorders and warrant further research [15,18]. Therefore, allopregnanolone and its agonists, such as brexanolone, should be studied in the future for the management of MDD along with related disorders.

## Review

Neurobiology of allopregnanolone

Allopregnanolone, also known as tetrahydroprogesterone, is a progesterone metabolite produced by the action of two enzymes in the brain, 5-reductase and 3-hydroxysteroid dehydrogenase. It acts as an allosteric modulator of the GABA-A receptor and exerts various physiological effects [19,20]. This neuroactive steroid (NAS) is metabolized by the liver and has a low bioavailability, thereby hindering its future use as a potential drug [19,21]. It is important to note that there exists a noteworthy decline in the concentrations of allopregnanolone during stress-induced circumstances, such as psychiatric conditions like depression and PPD, post-traumatic stress disorder (PTSD), anxiety disorders, and degenerative neurological diseases like Alzheimer’s disease (AD) and Parkinson’s disease (PD). A similar effect was noted in patients with multiple sclerosis (MS) as well [19]. This is further supported by the finding of increased allopregnanolone levels in the CSF due to the use of selective serotonin receptor inhibitors, as reported by many studies [22-24].

Brexanolone, an allopregnanolone agonist, has received approval for the treatment of PPD by the FDA [14,22]. Zuranolone, which is also an allopregnanolone agonist, has been studied as an antidepressant medication in different randomized controlled trials as an oral drug for PPD and MDD [22,25].

Role of Allopregnanolone in Neurophysiology and Neurosteroidogenesis

The major role of allopregnanolone is in the maturation of the fetal brain, including the development of neurons and the parts of the brain like the hippocampus, cerebral cortex, and thalamus. It plays a notable role in myelin formation in the CNS and in different feedback mechanisms modulating the gonadal axis, specifically luteinizing hormone-releasing hormone (LHRH). Studies have also suggested that the synthesis of neurotransmitters in the adult brain is allopregnanolone-dependent [19,26]. The unique role of modulating various ion channels and receptors, such as the GABAA receptor, contributes to its neuroprotective properties [14,27]. In addition to its neuroprotective effects, allopregnanolone has been reported to have antidepressant, anxiolytic, and analgesic effects [19,27-29].

Allopregnanolone's Impact on Mood Regulation

Allopregnanolone, also known as tetrahydropregnanolone, is a progesterone-derived NAS with direct paracrine effects on the CNS and peripheral nervous system (PNS) [19,30,31]. Allopregnanolone exerts sex-dimorphic effects in both the physiological and pathological lieu in males and females [19,30]. Recent studies have implicated higher concentrations of allopregnanolone and its isomers in female brains than in male brains. The sex-dimorphic action of allopregnanolone can be explained by the reduced concentration of this NAS in adult males [19]. However, the levels of NAS in similar adult females did not decrease. As allopregnanolone is a derivative of progesterone, its concentrations in the plasma, CNS, and PNS are altered by changes in gonadal hormones, such as menopause and pregnancy in females, and long-term post-gonadectomy in males [19].

Allopregnanolone regulates mood by direct and allosteric effects on GABAA receptors in the CNS. This, in turn, leads to a modification in the composition of GABAA receptor subunits [19,22]. This interaction with GABAA receptors holds great importance in mood regulation and thus implies altered levels of this NAS in multiple psychiatric conditions. Under physiological conditions, the direct effect of allopregnanolone mediated through GABAA receptors occurs even at very low concentrations, resulting in significant antidepressant, antistress, sedative, and anxiolytic effects [19,22]. Depression has been linked to lower levels of GABA and allopregnanolone when compared to healthy individuals. Studies have also revealed a relationship between altered levels of allopregnanolone, enzymes metabolizing it (i.e., reduced expression of 5-alpha reductase), and psychiatric disorders such as anxiety, postpartum anxiety, depression, and PPD [19]. PPD results from a rapid decline in hormones following parturition; therefore, it can be reversed by the administration of allopregnanolone [30]. Decreased plasma allopregnanolone levels are also implicated in PTSD [19]. Depressive symptoms, increased contextual fear, re-experiencing, and impaired extinction of fear, as part of PTSD, are all associated with reduced levels of plasma allopregnanolone.

Females with premenstrual dysphoric disorder (PMDD) [30] exhibit a reduced sensitivity of GABAA receptors to pregnanolone and diazepam, while their sensitivity to allopregnanolone is heightened. The negative mood symptoms experienced by these females can be attributed to the conflicting impact of allopregnanolone mediated through GABAA receptors [19,30]. In individuals with PMDD, low to moderate levels of allopregnanolone stimulate amygdala activity, leading to anxiety-like responses. Conversely, higher concentrations of allopregnanolone decrease amygdala activity in these individuals, inducing calming and anxiolytic effects similar to those achieved after benzodiazepine treatment. Therefore, PMDD in females can be attributed to low levels of progesterone and its metabolites, including allopregnanolone, unstable hormone levels, or altered receptors [30]. Another supporting evidence for the role of allopregnanolone in mood regulation is well-explained by post-finasteride syndrome (PFS) [19]. Finasteride is an anti-androgenic drug that inhibits 5-alpha reductase, which is vital for the synthesis of allopregnanolone. Studies have demonstrated a correlation between decreased levels of this particular enzyme and thus, diminished synthesis of allopregnanolone, with the emergence and expression of depressive symptoms.

In addition to mood regulation, allopregnanolone and other NASs play a significant role in brain maturation and pathological mechanisms underlying PD and AD [19]. Allopregnanolone and other NASs also have neuroprotective effects, such as in traumatic brain injury (TBI) and many inflammatory neurodegenerative diseases such as MS [32].

Allopregnanolone's Mechanism of Action to Treat MDD

Allopregnanolone, a NAS, and its agonists have opened new avenues for the management of MDD and related psychiatric conditions such as PMDD, PPD, bipolar disorder, and PTSD [14].

The various hypotheses that have been formulated to explain the pathogenesis of MDD include the following [22]: (a) the monoamine hypothesis, related to the depletion of monoamine neurotransmitters in the brain; (b) the glutamine hypothesis, which is associated with elevated glutamate levels; (c) the GABAergic deficit hypothesis, related to altered or defective GABAergic transmission in the brain.

Allopregnanolone and related NASs exert their effects by regulating GABAergic transmission in the brain by acting on the GABA-A receptors and thus have a significant effect on mood regulation [14]. GABAergic neurons are found abundantly in areas of the CNS that are particularly involved in regulating mood and emotions, memory and intelligence, as well as cognitive abilities, decision-making, sleep, pleasure, and motivation. These especially include interneurons and long projection neurons in the prefrontal cortex, amygdala, anterior cingulate cortex, ventral tegmental area, nucleus accumbens, and hippocampus [22]. Under physiological conditions, allopregnanolone alleviates depressive symptoms by allosteric modulation [29] of GABA-A receptors and by regulating the hypothalamic-pituitary-adrenal (HPA) axis, thereby maintaining physiological homeostasis [14]. Allopregnanolone regulates the HPA axis through its effect on corticotrophin-releasing hormone neurons, which are under the control of the δ subunit containing GABA-A receptors [19].

As GABAergic transmission has a significant role in the pathophysiology of MDD, it is imperative to understand the GABA-A receptors. GABA-A receptors are known to have 19 subunits: these include six α, three each of β, γ, and ρ, and one each of δ, ε, π, and θ [14]. However, the brain is rich in subunit combinations, including two α1, two β2, and one γ2 subunits, wherein the binding site for modulators is found at the junction between the α and β subunits [19]. Approximately 50 conformations exist naturally, with fewer conformations as pharmacological targets. Changes in the physiological conformation of these subunits have been found to be significant in the pathophysiology of MDD [14]. Allopregnanolone has a preference for action on the δ subunit of GABA-A receptors found only at extra-synaptic sites, unlike other GABA-A receptor agonists, such as benzodiazepines and barbiturates, which act only on intrasynaptic sites [14]. This explains why GABA-A, a pentameric chloride ion receptor, responds differently to different modulators and varies according to the composition of subunits. Furthermore, the GABA-A receptor subunit composition has been found to be altered by continuous exposure to progesterone and allopregnanolone, altering the plasticity of the GABA-A receptors; extra-synaptic neurons are most sensitive to allopregnanolone and other NASs [19].

Allopregnanolone exerts its effects on mood regulation by enhancing GABAergic transmission of chloride currents. Therefore, this NAS acts by hyperpolarizing postsynaptic membranes, causing inhibition of neurons, and hence leading to anxiolytic and anesthetic effects [30]. Another mechanism by which allopregnanolone modulates GABA-A receptor transmission is via phosphorylation of certain subunits of GABA-A receptor, thereby increasing the expression of GABA-A receptors on the cell surface [22]. Therefore, the above discussion implies that the altered constitution of GABA-A receptor subunits, reduced levels of allopregnanolone, and altered expression of enzymes regulating progesterone and allopregnanolone metabolism play roles in the pathophysiology of MDD [22]. Thus, allopregnanolone and other NASs regulate mood by fine-tuning neuronal inhibition via GABAergic transmission [30]. Under acute stress, allopregnanolone levels increase, indicating its neuroprotective effects. However, under chronic stress, such as in MDD, the allopregnanolone levels in the CNS are reduced, thereby altering GABAergic neuronal transmission, making it a likely target for the treatment of MDD [14].

Effective available treatments for MDD, such as SSRIs like fluoxetine, have been found to upregulate the brain stores of allopregnanolone and related NASs, enhancing GABAergic neuronal transmission, which is independent of the reuptake of serotonin and related monoamine neurotransmitters, which further implicates the role of allopregnanolone in the treatment of MDD [30]. The role of GABAergic transmission, allopregnanolone, and related NASs in MDD can be further supported by some findings in people who suffer from depression. These include diminished levels of GABA in the plasma, cerebral cortex, and cerebrospinal fluid (CSF), altered expression and subunit composition of GABA-A receptors, as well as reduced levels of NASs in the CSF of individuals suffering from depression [22]. Another advantage of allopregnanolone and other NASs used in the treatment of MDD is the faster onset of action compared to SSRIs and other antidepressant treatments, making it possible to act as a bridge between the two therapies and quicker improvement of depressive symptoms [14].

Allopregnanolone is also implicated in the pathophysiology of PMDD secondary to altered sensitivity of GABA-A receptors, with temporal variations over the ovarian cycle, to allopregnanolone and related NASs, leading to mood dysregulation [30,33,34]. Based on the altered expression of GABA-A receptor subunits, altered allopregnanolone levels are associated with PPD. A sudden drop in allopregnanolone levels after parturition is responsible for the manifestation of PPD [35]. Treatment of PPD with allopregnanolone analogs showed rapid improvement in PPD symptoms and was approved by the FDA [22]. Allopregnanolone plays an important role in fetal brain development and its levels sharply increase before parturition. It also plays a role in the development of the adolescent brain, adult behavior, and maturation of the nervous system [19]. The potential of allopregnanolone to inhibit MyD88-dependent toll-like receptors (TLRs) signal is key to the treatment of many pro-inflammatory neuronal diseases by halting the effects of TLR-activated inflammatory cytokines, chemokines, and interferons [32].

Allopregnanolone agonists

Brexanolone

Brexanolone is a preparation of allopregnanolone and comes in a soluble and injectable form [36]. It hence functions as a modulator of the GABA neurotransmitter system and mimics an intrinsic progesterone metabolite whose levels fluctuate during pregnancy and the postpartum period [37]. Even though many antidepressants are being used for the treatment of PPD, brexanolone is the first drug that got FDA approval for this condition. This has greatly escalated the interest in drug research and development [35]. The study characteristics of different randomized clinical trials (RCTs) conducted on the efficacy of brexanolone and zuranolone are shown in Table 1.

**Table 1:** Summary of RCTs on the efficacy of brexanolone and zuranolone for the treatment of depression Abbreviations: HAM-D = Hamilton Depression Rating Scale; MADRS = Montgomery–Åsberg Depression Rating Scale; BRX = brexanolone; LSM = least square mean; SE = standard error; MDD = major depressive disorder; PPD = postpartum depression; RCTs = randomized clinical trials.

Study ID	Sample size (intervention vs. placebo)	Dosage	Follow-up duration	Clinical diagnosis	Mean change in depression rating on HAM-D score post-treatment (intervention vs. placebo)	Response rate (% of patients) (reduction of >50% from baseline in HAM-D score)	Remission rate (a HAM-D score ≤ 7, MADRS ≤ 10)
Zuranolone							
Gunduz-Bruce et al. (2019) [38]	45 vs. 44	30 mg	4 weeks	MDD	-17.4 ± 1.3 vs. -10.3 ± 1.3	35 vs. 18	29 vs. 11
Deligiannidis et al. (2021) [25]	76 vs. 74	30 mg	30 days	PPD	-17.8 (1.04) (LSM (SE)) vs. -13.6 (1.07) (LSM (SE))	53 vs. 35	33 vs. 17
Clayton et al. (2023) [39]	159 (zuranolone 20 mg) vs. 166 (zuranolone 30 mg) vs. 157 (placebo)	20 mg and 30 mg	182 days	MDD	−11.5 (0.62) (LSM (SE)) (zuranolone 20 mg) vs. −12.5 (0.68) (LSM (SE)) (zuranolone 30 mg) vs. −11.1 (0.59) (LSM (SE))	65 (zuranolone 20 mg) vs. 77 (zuranolone 30 mg) vs. 60 (placebo)	35 (zuranolone 20 mg) vs. 48 (zuranolone 30 mg) vs. 33 (placebo)
Clayton et al. (2023) [40]	266 vs. 268	50 mg	28 days	PPD	−14.1 (0.51) (LSM (SE)) vs. −12.3 (0.50) (LSM (SE))	139 vs. 118	74 vs. 68
Kato et al. (2023) [41]	85 (zuranolone 20 mg) vs. 82 (zuranolone 30 mg) vs. 82 (placebo)	20 mg and 30 mg	12 weeks	MDD	-8.14 ± 0.62 (zuranolone 20 mg) vs. -8.31 ± 0.63 (zuranolone 30 mg) vs. -6.22 ± 0.62 (placebo)	18 (zuranolone 20 mg) vs. 25 (zuranolone 30 mg) vs. 13 (placebo)	8 (zuranolone 20 mg) vs. 7 (zuranolone 30 mg) vs. 3 (placebo)
Brexanolone							
Kanes et al. (2017) [42]	10 vs. 11	30-90 μg/kg/hour	30 days	PPD	-21 (2.9) (LSM (SE)) vs. -8.8 (2.8) (LSM (SE))	8 vs. 3	7 vs. 1
Meltzer-Brody et al. (2018) - Trial 1 [43]	45 (BRX90) vs. 47 (BRX60) vs. 46 (placebo)	30-90 μg/kg/hour	30 days	PPD	−17.7 (1.2) (LSM (SE)) (BRX90) vs. −19.5 (1.2) (LSM (SE)) (BRX60) vs. -14.0 (1.1) (LSM (SE)) (placebo)	-	12 (BRX90) vs. 19 (BRX60) vs. 7 (placebo)
Meltzer-Brody et al. (2018) - Trial 2 [43]	54 vs. 54	30-90 μg/kg/hour	30 days	PPD	-14.6 (0.8) (LSM (SE)) vs. -12.1 (0.8) (LSM (SE))	-	30 vs. 20

Role in PPD: PPD is a highly widespread psychiatric disorder that poses a negative impact on the long-term health of both mothers and their children [35]. Four clinical trials, including three RCTs and one quasi-randomized study, have been published on the efficacy of brexanolone and its role in PPD. A meta-analysis by Zheng et al. [44] reported results from three RCTs and found that it showed greater response rates (RR = 1.50; 95% CI, 1.06-2.13) and remission rates (RR = 2.20; 95% CI, 1.31-3.70) than the control group. The adverse drug reactions were similar between groups.

Safety: Brexanolone causes adverse effects like somnolence, dizziness, and headache. Moreover, the need for continuous oxygen saturation monitoring, administration as a continuous infusion, and the requirement of an inpatient facility are some of the common drawbacks of brexanolone infusion [45]. Due to the side effects of somnolence and dizziness, it is suggested that patients avoid any tasks that would require them to be highly attentive, such as driving. It is advised to avoid the use of brexanolone in patients suffering from end-stage renal disease (estimated glomerular filtration rate <1 5/min/1.73 m2) because their kidneys are unable to remove a substance called betadex sulfobutyl ether sodium, which causes further damage to the kidneys. However, patients with only mild to moderate renal damage can take the normal dose of brexanolone.

Brexanolone is an inhibitor of CYP2C9, so caution should be exercised before administering any drug that is metabolized by this cytochrome p450 enzyme [37]. Brexanolone represents a significant development in the treatment of PPD and provides a targeted approach to addressing this debilitating condition. It can be considered superior to other drugs for PPD owing to its rapid onset of action [46].

Zuranolone

Zuranolone, another synthetic NAS, is a novel drug whose efficacy has recently been studied. Five RCTs have been published on the role of zuranolone in the treatment of PPD and MDD. The results of a double-blind phase II trial on patients with MDD by Gunduz-Bruce et al. showed that 79% of patients in the zuranolone group had an initial response compared with 41% of patients in the placebo group (OR: 9.6; 95% CI: 2.9 to 31.6) [38]. Consequently, remission rates were also reported in this study and were higher than expected for the zuranolone group (64%) compared to the placebo group (26%) (OR: 5.3, 95% CI: 2.1 to 13.3), and the Hamilton Depression Rating Scale (HAMD-17) score was 7.

The MOUNTAIN study is a phase 3, multicenter, double-blind, randomized, placebo-controlled trial in which the patients were randomized into groups of once-daily zuranolone 20 mg, zuranolone 30 mg, and placebo. Unexpectedly, the study did not meet the primary endpoint (HAMD-17 score change from baseline on day 15) for the zuranolone group vs. the placebo group (least squares mean difference from placebo = -1.4; p = 0.115). However, a rapid onset of symptom improvement from baseline was reported on days three, eight, and 12, and even up to day 15 in the 30 mg group. On day 42, a change from the baseline in the mean HAMD-17 score was sustained in this group [39]. Individuals who completed extended monitoring over a period of six months demonstrated sustained enhancement of symptoms, setting zuranolone as a drug with potential importance in all upcoming MDD treatment regimens [47]. According to the most recent phase 3 RCT by Clayton et al. [39], zuranolone was associated with a statistically significant improvement in depressive symptoms (measured by the change from baseline HAMD scores) on days three and 15. This effect was sustained at all the follow-up visits.

Safety and tolerability consideration: In a phase II trial conducted by Gunduz-Bruce et al., at least 53% of patients reported at least one adverse effect compared to the placebo group, in which the occurrence was 45% [38]. However, there was a dose-dependent increase in side effects in the MOUNTAIN trial, with the 30 mg group experiencing more side effects (54.2%) than the placebo group. Other side effects similar to those in the phase II trial included somnolence, diarrhea, nausea, and exhaustion. The fact that there was no increase in suicidal ideation from baseline is crucial [39].

Ganaxolone

Ganaxolone belongs to a group of synthetic analogs of the natural neurosteroid allopregnanolone. Unlike benzodiazepines, which specifically bind to GABAA receptors, ganaxolone has an affinity for GABAA receptors that contain d-subunits. GABAA receptors have been the main target of several antiseizure drugs (ASDs) [48]. Numerous investigations have provided evidence of the effectiveness of ganaxolone in various patients diagnosed with epilepsy [49]. Current research and development plans include phase 2 and phase 3 trials aimed at exploring alternative therapeutic applications for ganaxolone, such as PPD, pharmacoresistant status epilepticus, as well as certain rare genetic epilepsies that do not respond to traditional treatments like CDLK5 deficiency disorder. Researchers are focusing on ganaxolone as a potential treatment option specifically designed to address cases of pharmacoresistant status epilepticus [50].

SGE-516

SGE-516, a NAS, serves as a positive allosteric modulator of the gamma and delta subunits found in GABAA receptors. Its widespread GABAA receptor action sets it apart from benzodiazepines, which have a specific affinity for GABAA receptors, including the gamma subunit. SGE-516 can be used for long-term oral dosing [51]. In preclinical models of PPD, the efficacy of SGE-516 in reducing depressive behaviors and enhancing maternal care has been demonstrated. It also suppresses the activation of the stress-induced HPA axis [50].

Challenges and limitations

Brexanolone and zuranolone are examples of allopregnanolone agonist treatments that have demonstrated potential in the management of depressive disorders. However, they have some limitations, similar to those of any medical intervention. The potential drawbacks of allopregnanolone agonist treatment for depressive disorders are mentioned below.

Variability in Treatment Response

Many underlying causes and symptoms have been associated with depressive disorders. Variations in the exact type and subtype of depression may cause variability in treatment response. Each person has a different brain chemistry, which might affect their response to certain medicines or therapies. Additionally, individual differences in how allopregnanolone agonists interact with GABAA receptors can affect the response of patients to treatment. Genetic differences can affect the function of GABAA receptors and other neurotransmitter systems. Some people may respond differently to allopregnanolone agonist therapy depending on specific hereditary variables. The ideal length and dosage of allopregnanolone agonist therapy are still under investigation. Individual variations in the treatment response can be influenced by the precise dosage and duration of treatment. Anxiety disorders and substance use disorders frequently coexist with depressive disorders, among other mental health issues. These coexisting conditions may affect the patient’s response to treatment and increase the unpredictability of results. The placebo effect may also have an impact on the treatment's effectiveness. Their subjective sense of progress may have been influenced by their expectations and opinions regarding the effectiveness of treatment.

Side Effects and Safety Concerns

Zuranolone has a good safety profile according to many RCTs [25]. It is associated with fatigue, headache, somnolence, dizziness, diarrhea, nausea, and sedation. Two patients experienced hypomania [14]. One patient experienced confusion while undergoing zuranolone treatment [25]. Fatigue, dizziness, somnolence, and headache are the prevailing adverse effects reported with brexanolone. Some individuals have experienced more severe complications like increased sedation and even loss of consciousness [52]. One participant was noted to have suicidal ideation and attempted an overdose, whereas another participant experienced sinus tachycardia [42]. Severe insomnia has also been noted in another study participant [14]. Additionally, brexanolone should be used with caution in pregnant patients and end-stage kidney disease patients due to decreased renal clearance of its solubilizing agent, as well as interaction with the cytochrome P-450 system in the liver [37], as well as the potential for abuse in recreational drug users as a dependent euphoric mood was observed [46].

Brexanolone is administered intravenously (IV). The need for physician supervision and constant pulse oximetry monitoring during the 60-hour intravenous infusion in a clinical context can be inconvenient for some patients and may prevent them from receiving therapy [46]. Additionally, it is essential for physicians to closely observe and assess patients every two hours in search of indications of over-sedation [53]. Although the oral administration of zuranolone is more practical, it still requires a consistent dosage and adherence to the recommended schedule. It was administered once daily as an oral pill for two weeks [45]. However, there are numerous advantages, such as greater effectiveness, faster response times, and remission of disease, which can be used in individuals with a limited response to psychotherapy or serotonin reuptake inhibitors [46]. Furthermore, the duration of treatment is specified (60 hours for brexanolone and 15 days for zuranolone), thereby reducing the pill burden [14].

Clinical Trial Design Considerations

There is an ethical consideration that some patients were administered a placebo instead of an active new drug, which could potentially have a positive effect on their disease. Moreover, individuals undergoing medical treatment might experience added stress due to the prolonged duration of hospitalization and frequent travel required to attend various appointments at the research center [54]. It is difficult to generalize the results, as the studied population is very different from the actual population treated [53]. Clinically significant placebo response rate was identified as a confounding variable [39]. The effects of allopregnanolone agonists on lactation have not been studied because of the exclusion criteria for breastfeeding women [25]. Some studies had a limited follow-up duration of only 30 days, which is insufficient for adequate surveillance [46]. The sample size was small in some studies [53].

Future directions and potential applications

Combination Therapies and Augmentation Strategies

The efficacy of specific drug combinations is under investigation in the treatment of MDD. One trial specifically aimed to target the endogenous opioid system. Results from various studies show that a combination therapy comprising buprenorphine, samidorphan, and an antidepressant demonstrated superiority over placebo combined with antidepressants in certain cases but not consistently across all studies. However, additional research is necessary to deepen our understanding of this field [55]. AXS-05 is another drug, currently being investigated, which contains low-dose bupropion and dextromethorphan. This combination drug exerts its therapeutic effects through multiple mechanisms of action. These include non-competitive antagonism at the NMDA receptor, agonism at sigma-1 receptors, antagonism at nicotinic acetylcholine receptors, as well as inhibition of serotonin, noradrenaline, and dopamine transporters [56]. In August 2022, AXS-05 obtained clearance from the FDA for the treatment of MDD after successfully completing two phases of clinical trials. The results showed that AXS-05 demonstrated superior efficacy compared to low-dose bupropion or a placebo [56]. An additional study investigated the effectiveness of transdermal estradiol in combination with intermittent micronized progesterone in preventing depressive symptoms among peri-menopausal and early post-menopausal women who were initially euthymic (without any mood disturbances). The findings indicated that this hormonal intervention was able to prevent the onset of such symptoms [57]. In a phase 2 RCT, pimavanserin, which functions as an antagonist for the 5-HT2A receptor, exhibited promising outcomes when used in conjunction with standard treatment methods for MDD. However, subsequent research conducted via phase 3 clinical trials failed to identify any significant improvement compared to the use of a placebo [58]. Another recent clinical trial found that pioglitazone, which acts as an agonist of the peroxisome proliferator-activated receptor gamma along with citalopram and chlordiazepoxide combination therapy, was more effective than a placebo [58]. Furthermore, in another phase 3 trial, it has been shown that co-initiating zuranolone at a dosage of 50 mg/day with traditional antidepressants leads to significantly better results than using a placebo. This finding supports the notion that combining this drug with conventional antidepressant medications can optimize its efficacy rate [59]. Thus far, there have been ongoing efforts dedicated toward identifying additional combinations of drugs for treating MDD.

Allopregnanolone Agonists in Other Diseases

Allopregnanolone agonists exhibit remarkable antidepressant properties and have also been recognized for their therapeutic benefits in other conditions. Numerous trials have examined the effectiveness of ganaxolone for refractory epilepsy. A study shows a 50% reduction in seizure frequency from baseline to the double-blind phase, but further long-term RCTs are required because there are very few relevant studies [60].

Implications for research and clinical practice

Allopregnanolone agonists have shown promise in the management of depressive disorders, particularly in individuals who do not respond well to conventional therapies. More studies are needed to fully understand the mechanisms underlying the efficacy of these agonists and their potential side effects. Future research could provide valuable information about the neurobiological basis of depressive disorders, and potentially pave the way for the development of more targeted and effective treatments. Furthermore, the use of allopregnanolone agonists may have implications in clinical practice. In terms of clinical practice, incorporating allopregnanolone agonists into treatment protocols for depressive disorders holds promise for improving outcomes. These agonists could be considered as an alternative or adjunctive therapy for patients who have not achieved adequate symptom relief with standard treatments. Based on the currently existing published data, there is a lack of clarity regarding whether having a previous history of MDD has an impact on the likelihood of experiencing a response or remission. There are many ongoing clinical trials on the efficacy of brexanolone for various depressive disorders such as PTSD. Future research should concentrate on separating postpartum recurrence of MDD from new-onset or recurrent PPD [14].

## Conclusions

Brexanolone is the first allopregnanolone agonist licensed by the FDA for the treatment of PPD. It works via the positive allosteric modulation of GABAA receptors. However, because it requires an infusion of over 60 hours and a minimum inpatient stay of two to five days, its usage is restricted. This poses the risk of interference with breastfeeding, childcare, and early attachment. Brexanolone poses significant risks of drowsiness and loss of consciousness, necessitating close patient monitoring. However, zuranolone (an oral allopregnanolone agonist that has not yet received FDA approval) has performed better in RCTs for the treatment of PPD and MDD (at a higher dose than that of PPD), with respect to response time, a low overall incidence of side effects, and the absence of significant drug-drug interactions.

Brexanolone's effectiveness in treating MDD has to be further investigated. Clinical trials demonstrating the effectiveness of brexanolone and zuranolone in treating depression that is resistant to medication are also required, given that one-third of patients with MDD continue to be treatment-resistant.
